# Cost and health‐related quality of life for children hospitalized with respiratory syncytial virus in Central China

**DOI:** 10.1111/irv.13180

**Published:** 2023-08-17

**Authors:** Lingshuang Ren, Lidan Cui, Qianli Wang, Liujiong Gao, Meng Xu, Meng Wang, Qianhui Wu, Jinxin Guo, Li Lin, Yuxia Liang, Nuolan Liu, Yibing Cheng, Juan Yang, Hongjie Yu

**Affiliations:** ^1^ School of Public Health Fudan University, Key Laboratory of Public Health Safety, Ministry of Education Shanghai China; ^2^ Henan Engineering Research Center of Pediatric Infection and Critical Care Children's Hospital Affiliated to Zhengzhou University Zhengzhou China; ^3^ Shanghai Institute of Infectious Disease and Biosecurity Fudan University Shanghai China

**Keywords:** children, cost, health‐related quality of life, hospitalization, respiratory syncytial virus

## Abstract

**Background:**

The economic burden of respiratory syncytial virus (RSV) infection and its impact on health‐related quality of life (HRQoL) are not well‐understood in China. This study assessed total cost and HRQoL for children hospitalized with RSV in Central China.

**Methods:**

Based on a prospective case series study in Henan Province in 2020–2021, inpatients aged 0–59 months with RSV‐related acute respiratory infections (ARIs) were included into analysis. Total cost included direct medical cost (sum of medical cost before and during hospitalization), direct non‐medical cost, and indirect cost. Direct medical cost during hospitalization data were extracted from the hospital information system. Other costs and HRQoL status were obtained from a telephone survey conducted in the caregivers of the enrolled patients.

**Results:**

Among 261 RSV‐infected inpatients, caregivers of 170 non‐severe cases (65.1%, 170/261) were successfully interviewed. Direct medical cost per episode was 1055.3 US dollars (US$) (95% CI: 998.2–1112.5 US$). Direct non‐medical cost and indirect cost per episode were 83.6 US$ (95% CI: 77.5–89.7 US$) and 162.4 US$ (95% CI: 127.9–197.0 US$), respectively. Quality adjusted life years (QALY) loss for non‐severe RSV hospitalization was 8.9 × 10^−3^ (95% CI: 7.9 × 10^−3^–9.9 × 10^−3^). The majority of inpatients were <1 year of age comprising significantly higher cost and more QALY loss than older children.

**Conclusions:**

RSV‐associated hospitalization poses high economic and health burden in Central China particularly for children <1 year old. Our findings are crucial for determining the priority of interventions and allocation of health resources.

## 1 INTRODUCTION

Respiratory syncytial virus (RSV) is a leading cause of hospitalization for young children with acute lower respiratory tract infection, resulting in a considerable disease burden around the world.^1^ With 33.0 million RSV‐associated acute lower respiratory infection episodes, 3.6 million hospitalizations, and 26,300 in‐hospital deaths in children aged 0–59 months in 2019,^2^ it was estimated that RSV accounts for about 28% of all acute lower respiratory tract infection episodes and 13%– 22% of acute lower respiratory infection related mortalities globally.^3^ Additionally, RSV infection creates a substantial economic burden across the globe.^4^ The global cost for inpatient management was estimated to be €4.82 billion in 2017 from the healthcare payers' perspective.^5^

Several candidates for RSV vaccines, immunoprophylaxis, and immune therapeutics are in clinical trials.^6^ Two available preventive measures—palivizumab and polyclonal RSV intravenous immunoglobulin (RSV‐IVIG) product—are primarily recommended for children at high risks but not widely used due to higher cost.^5^, ^7^ Nirsevimab was recently approved in Europe for the prevention of RSV lower respiratory tract disease in neonates and infants during their first RSV season.^8^ Estimations of RSV‐related health and economic burden are required for assessing the potential benefits of introduction of these products in countries and regions where no such interventions have been licensed yet, including China.^9^ However, there are limited numbers of studies assessing the economic burden and health‐related quality of life (HRQoL) associated to RSV infection in China with most of the studies evaluating only the direct medical cost of hospitalization.^10^

In the present work, we conducted a prospective study in Central China to estimate the total cost and assess the HRQoL in children hospitalized with RSV‐associated acute respiratory infection (ARI).

## 2 METHODS

### 2.1 Study design and population

This study was based on a prospective case series study conducted at Henan Children's Hospital, the largest tertiary pediatric hospital in Henan province of Central China, between December 29, 2020 and October 20, 2021. Inpatients, aged under 5 years and having an acute onset of symptoms of respiratory infection, with or without fever (depending on whether aged over 3 months or not), were identified as ARIs cases and enrolled. The respiratory specimens from the participants were tested for RSV by real‐time reverse transcription polymerase chain reaction (real‐time RT‐PCR) within 48 h of admission. Detailed study design and enrollment process were described in the previous study.^11^

### 2.2 Data collection and telephone survey

General characteristics collected for the participants included demographics (age, gender, address, etc.), underlying conditions (preterm birth, congenital diseases, airway dysplasia, etc.), medical history (date of onset, date of hospital admission, date of discharge, etc.), and clinical outcomes (mechanical ventilation, intensive care unit [ICU] admission, complications, etc.). These data were extracted directly from the hospital information system (HIS) and checked for completeness and accuracy by the investigators.

The total cost described in this study included direct medical cost, direct non‐medical cost, and indirect cost throughout the whole RSV infection episode (Figure S1). Direct medical cost was the sum of direct medical cost during and before the hospitalization including cost for examinations, treatment, consultation charges (ward and outpatient consultation), self‐medication, and so forth.^12^ Direct non‐medical cost included cost of transportation, food, and accommodation for the patients and caregivers. Indirect cost accounted for the caregivers' loss of income while taking care of the patients. The direct medical cost during hospitalization was obtained from HIS including both reimbursable charges (covered by medical insurance) and the out‐of‐pocket expenses. The direct medical cost prior to hospitalization, direct non‐medical cost, indirect cost, and the HRQoL status were obtained from the telephone survey.

The telephone survey was conducted between January 20 and November 19, 2021. Caregivers of the enrolled patients were called by a trained nurse, under the supervision of the research investigators, after ≥14 days of discharge. Each telephone number was dialed three times on different days before classifying them as unreachable. After obtaining verbal consent, the caregivers were requested to provide information related to cost and HRQoL for the corresponding RSV episode. The questionnaire covered the following topics: (i) disease‐related information (e.g., how long the symptoms persisted); (ii) direct medical cost prior to hospitalization (e.g., whether visited healthcare facilities before hospitalization, the amount of direct medical expenditure prior to hospitalization, etc.); (iii) direct non‐medical cost attributable to the RSV episode; (iv) indirect cost (e.g., the loss of workdays for caregiving); (v) HRQoL of the patients during the episode. If the interviewees were unable to recall quantitative information, for example, medical cost before admission or food cost per day, they were asked to provide a range of cost. The brief version of questionnaire can be seen in Supporting Information.

The Pediatric Quality of Life Inventory™ (PedsQL™) 4.0 Generic Core Scales (GCS) and Infant Scales modular instruments were used in the survey to measure HRQoL of patients during RSV hospitalization.^13^, ^14^ GCS were applicable to children ≥2 years old and Infant Scales were used for younger children under 24 months. PedsQL™ consists of four scales: physical functioning, emotional functioning, social functioning, and cognitive (for Infant Scales) or school functioning (for GCS) over the previous month. On a scale ranging from 0 to 100, the higher the score was, the better quality of life had been.

The general characteristics and direct medical cost during hospitalization data were also collected from the HIS for the patients who were unreachable in the telephone survey to examine if there had been any notable differences between the patients who were successfully interviewed and those who were not. In addition, we retrospectively extracted general characteristics and direct medical cost during hospitalization data for 386 RSV‐infected inpatients admitted in 2018–2019 using the same procedure to explore if variations existed in direct medical cost during hospitalization between the years due to the circulation of different subgroups or genotypes of RSV.^15^, ^16^

### 2.3 Data analysis

Data analyses were performed in R (version 4.1.2, R Foundation for Statistical Computing, Vienna, Austria). All costs were described as mean with 95% confidence interval (95% CI).^17^ Cost spent before 2021 were inflated to the equivalent of 2021 Chinese Yuan (CNY) using the consumer price index for China,^18^, ^19^, ^20^ and then were converted to US dollar (US$) according to the average exchange rate of 2021 (1 US$ = 6.45 CNY). General characteristics and costs were also described by two subgroups based on the severity of illness. Severe RSV cases was defined as those with illness that requiring ICU admission, different types of ventilation support during the hospitalization, or in‐hospital death.^21^

The total cost per RSV episode was the sum of direct medical cost, direct non‐medical cost and indirect cost of patients who completed the telephone survey. The direct medical cost was calculated by adding up the direct medical cost during hospitalization and prior to hospitalization of each case. Indirect cost was calculated using the human capital method—by multiplying mean daily income per capita with the number of days the caregivers were absent from the work during the episode.^22^

Generalized linear models (GLMs) were performed to explore potential risk factors associated with aforementioned types of cost. At first, the costs were logarithmically transformed. Then a univariate analysis was done on them to identify potential factors correlated to the cost. Finally, a forward stepwise model selection was performed using Akaike's Information Criterion (AIC) and likelihood ratio test to select the explanatory variables in multivariate analyses. A *p*‐value of <0.05 was considered statistically significant.

PedsQL™ scores could not be used to calculate a quality adjusted life years (QALY) outcome directly as it is a non‐preference‐based measure (NPBM) instrument.^23^ Therefore, a mapping algorithm was adopted to convert the scores of PedsQL™ to the utility score of the EQ‐5D‐Y (see Supporting Information).^24^ Finally, the loss of QALY was estimated by multiplying the difference of health utility during the RSV episode and full health (assuming “full health” utility = 1.0)^25^ with the reported duration of RSV illness.

### 2.4 Sensitivity analysis

As mentioned before, if a range of cost was obtained in the survey (instead of exact cost), the midrange value was taken in the baseline analysis to evaluate the total cost related to RSV hospitalization. Then sensitivity analyses were conducted by using the lower and upper limits of the ranges to test the impact of uncertainty on the results.

## 3 RESULTS

### 3.1 General characteristics of patients

**FIGURE 1:**
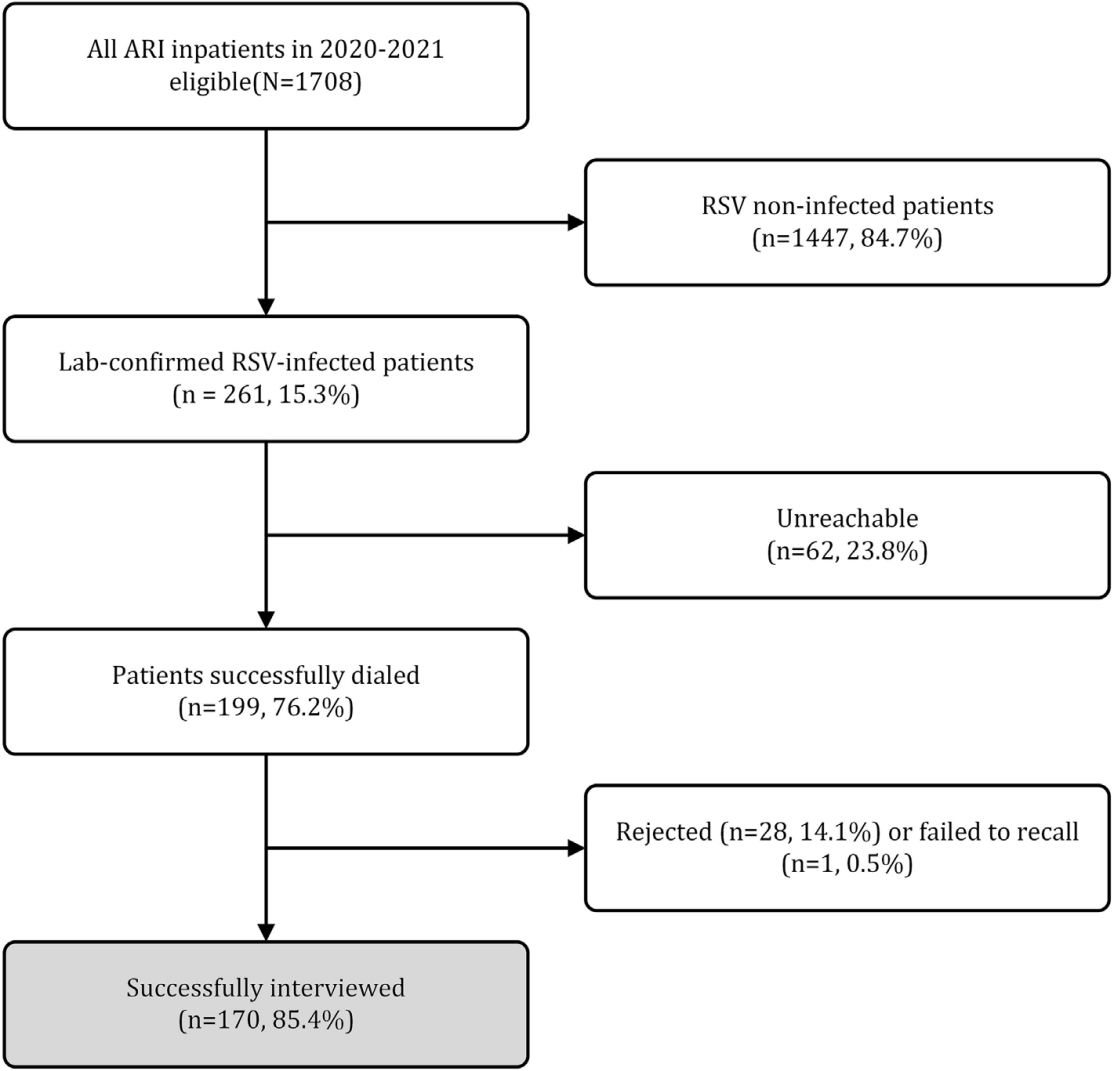
Flow chart for the enrollment RSV‐infected inpatient for total cost and health‐related quality of life (HRQoL) analysis.

A total of 261 (15.3%) laboratory‐confirmed RSV cases were identified among 1708 ARI patients aged 0–59 months. Among 261 candidates targeted for telephone survey, 62 (23.8%, 62/261) cases were unreachable (call unanswered for three times or telephone number missing). Out of the remaining 199 (76.2%) cases, 28 (14.1%) refused to participate in the interview and 1 (0.5%) failed to remember cost of the RSV episode. Overall, interviews were successfully performed for 170 cases achieving a completion rate of 65.1% (170/261) (Figure 1).

The overall basic characteristics of 261 RSV‐infected inpatients are shown in Table 1. The majority of patients were at 0–11 months of age (58.2%) and male (58.2%). The vast majority of patients were without underlying conditions (92.7%), that is, otherwise healthy, and for patients <1 year the proportion was 91.4% (139/152). General characteristics of the 170 successfully interviewed and the 91 excluded patients were comparable. However, all the cases that were successfully interviewed was non‐severe cases (*p* = 0.005). Only five severe cases were identified, among whom four were under 1 year old (Table S1).

**TABLE 1:** Characteristics of successfully interviewed and excluded patients.

Characteristic	Overall	Interviewed	Excluded^†^	*p*‐Value
	(*n* = 261)	(*n* = 170)	(*n* = 91)	
Age; month, median (IQR)	7.0 (2.7–18.0)	6.2 (2.6–17.0)	10.6 (3.0–18.0)	0.069
Age group				0.056
0–11 months	152 (58.2)	107 (62.9)	45 (49.5)	
12–23 months	62 (23.8)	33 (19.4)	29 (31.9)	
24–59 months	47 (18.0)	30 (17.6)	17 (18.7)	
Gender				0.293
Male	152 (58.2)	103 (60.6)	49 (53.8)	
Female	109 (41.8)	67 (39.4)	42 (46.2)	
Residence				0.759
Zhengzhou City	214 (82.0)	141 (82.9)	73 (80.2)	
Outside Zhengzhou City in Henan Province	43 (16.5)	26 (15.3)	17 (18.7)	
Outside Henan Province	4 (1.5)	3 (1.8)	1 (1.1)	
Ward				0.034
Infant Ward	123 (47.1)	87 (51.2)	36 (39.6)	
General Emergency Ward	136 (52.1)	83 (48.8)	53 (58.2)	
PICU	2 (0.7)	0 (0)	2 (2.2)	
Underlying condition				0.492
No	242 (92.7)	159 (93.5)	83 (91.2)	
Yes^‡^	19 (7.3)	11 (6.5)	8 (8.8)	
Complications				>0.999
No	3 (1.1)	2 (1.2)	1 (1.1)	
Yes^§^	258 (98.9)	168 (98.8)	90 (98.9)	
Severe illness	5 (1.9)	0 (0)	5 (5.5)	0.005
Length of hospital stay; median (IQR)	6.0 (5.0–7.0)	6.0 (5.0–7.0)	6.0 (5.0–7.0)	0.964

*Note:* Figures are numbers of patients (%) unless stated otherwise. Characteristics are compared between patients interviewed and excluded. A *p*‐value of <0.05 was considered statistically significant.

Abbreviation: IQR, interquartile range; PICU, pediatric intensive care unit.

^†^ The reasons for exclusion include unreachable, rejecting to participate or failed to recall cost of the RSV episode.

^‡^ Mainly including preterm birth, congenital heart diseases, airway dysplasia, asthma and other congenital or chronic comorbidities.

^§^ Mainly including pneumonia, bronchitis, respiratory failure, hepatic function abnormality, myocardial injury, and sepsis.

### 3.2 Total cost of patients hospitalized with RSV

Table 2 summarizes direct medical, direct non‐medical, and indirect cost for the 170 non‐severe RSV patients who were successfully interviewed. The mean direct medical cost was 1055.3 US$ (95% CI: 998.2–1112.5 US$). There was no significant difference in direct medical cost between patients aged 0–5 and 6–11 months (Table S2). Direct medical cost was significantly higher for patients aged 0–11 months than that for the older ones. Overall, 90.9% of the direct medical cost was spent during hospitalization (959.3 US$, 95% CI: 921.4–997.3 US$) (Table S3).

**TABLE 2:** Direct medical, direct non‐medical, and indirect cost of non‐severe RSV‐infected inpatients.

Characteristics	Direct medical cost	*p*‐Value	Direct non‐medical cost	*p*‐Value	Indirect cost	*p*‐Value
Overall	1055.3 (998.2–1112.5)		83.6 (77.5–89.7)		162.4 (127.9–197.0)	
Age group		<0.001		0.003		0.666
0–11 months	1152.7 (1074.5–1230.9)		91.3 (83.1–99.5)		163.6 (118.7–208.6)	
12–23 months	903.5 (799.2–1007.7)		66.7 (57.6–75.9)		147.0 (68.5–225.4)	
24–59 months	875.3 (809.0–941.5)		74.8 (61.0–88.7)		175.0 (93.2–256.8)	
Gender		0.564		0.909		0.277
Male	1074.7 (994.0–1155.4)		83.7 (75.7–91.8)		163.9 (123.4–204.3)	
Female	1025.6 (948.6–1102.5)		83.4 (73.9–92.8)		160.2 (97.0–223.4)	
Insurance		0.507		0.669		0.157
No	1120.8 (969.6–1272.0)		83.1 (69.4–96.9)		158.2 (73.9–242.4)	
Yes	1032.5 (975.2–1089.7)		83.8 (77.0–90.5)		163.9 (126.9–200.9)	
Underlying conditions		0.077		0.019		0.483
No	1048.1 (988.4–1107.9)		82.1 (75.8–88.5)		157.7 (123.1–192.3)	
Yes	1159.7 (950.9–1368.5)		104.6 (84.8–124.4)		230.7 (14.4–447.0)	
Complications		0.186		0.230		0.873
No	803.0 (503.4–1102.6)		54.3 (−164.6–273.2)		142.3 (−256.8–541.3)	
Yes	1058.3 (1000.7–1116.0)		83.9 (77.8–90.1)		162.7 (127.7–197.6)	

*Note*: Figures are mean (95% CI) of cost.

The mean direct non‐medical cost and indirect cost for non‐severe RSV hospitalization were 83.6 US$ (95% CI: 77.5–89.7 US$) and 162.4 US$ (95% CI: 127.9–197.0 US$), respectively (Table 2). Direct non‐medical cost between patients aged 0–5 and 6–11 months also showed no significant difference (Table S2). Significantly higher direct non‐medical cost was associated with patients aged 0–11 months than older age groups. Moreover, patients with underlying conditions were likely to pay more direct non‐medical expenses than those without (*p* = 0.019).

The mean total cost per RSV episode was 1301.4 US$ (95% CI: 1230.7–1372.0 US$). Direct medical cost accounted for 81.1% of the total cost (1055.3 US$/1301.4 US$), followed by indirect cost (12.5%, 162.4 US$/1301.4 US$), and direct non‐medical cost (6.4%, 83.6 US$/1301.4 US$). Total cost of patients under 1 year was significantly higher than that of those aged 1–5 years.

**FIGURE 2:**
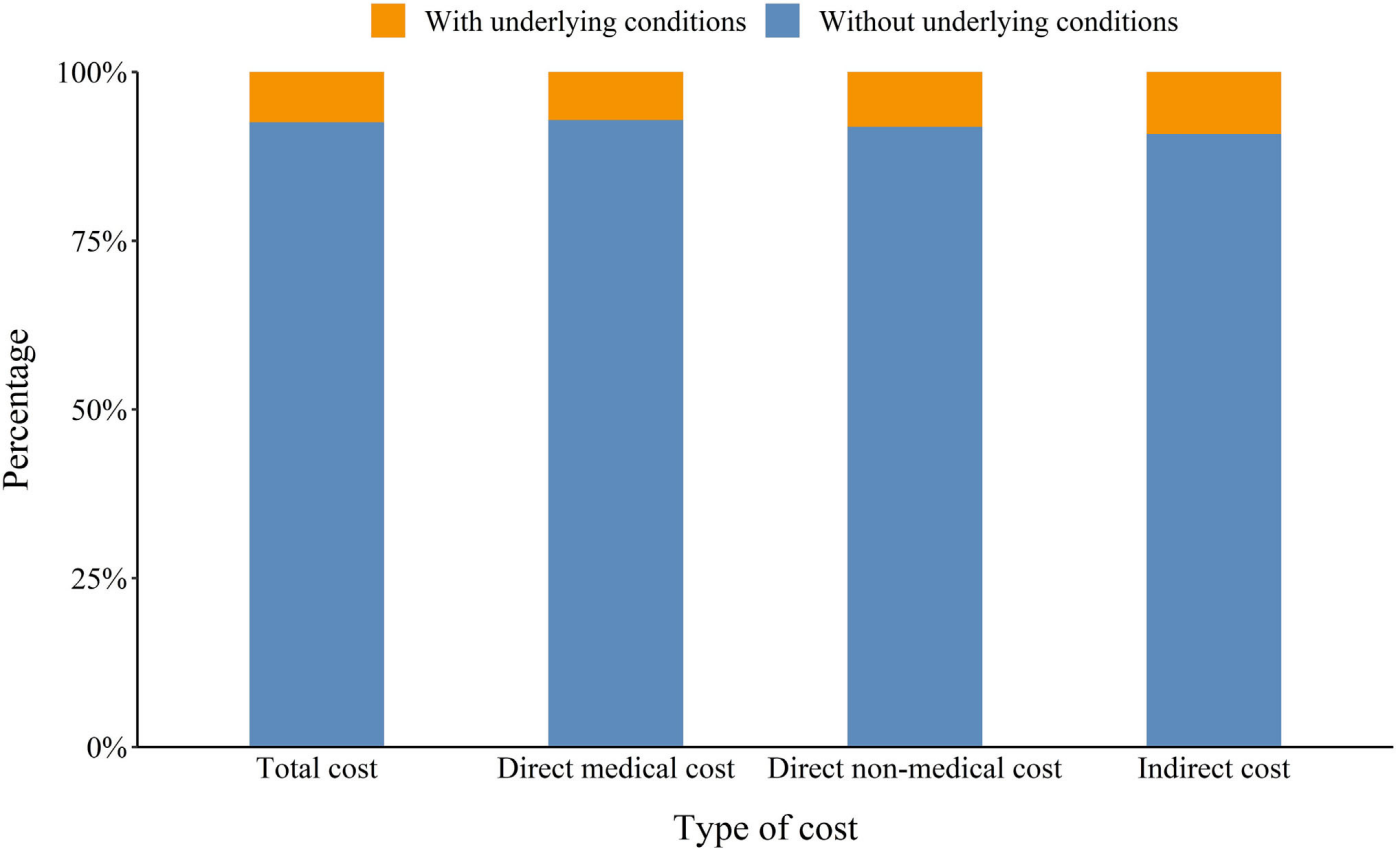
Proportion of cost for RSV‐infected inpatients with or without underlying conditions (overall 170 patients were successfully interviewed; 159 patients were without underlying conditions and 11 patients had underlying conditions).

However, the sum of total cost for cases without underlying conditions made up 92.6% of that for all 170 non‐severe patients who were successfully interviewed. With respect to direct medical, direct non‐medical and indirect cost, the proportions were 92.9%, 91.9%, and 90.8%, respectively (Figure 2).

### 3.3 Comparison of medical cost during hospitalization

The direct medical cost during hospitalization of all 261 RSV‐infected patients are presented in Table S3. The mean direct medical cost during hospitalization of all 261 RSV‐infected patients (1065.1 US$; 95% CI: 925.0–1205.1 US$) was higher than that for 170 patients who were successfully interviewed. This is justified as the five severe cases—who could not be interviewed—paid much higher than non‐severe cases (7207.8 US$; 95% CI: 82.7–14333.0 US$ vs. 945.1 US$; 95% CI: 911.3–978.8 US$) (*p* < 0.001). The out‐of‐pocket portion of direct medical cost during hospitalization was 764.9 US$ (95% CI: 636.2–893.6 US$) constituting 71.8% of the overall direct medical cost during hospitalization. There was no significant difference in the direct medical cost during hospitalization between patients who were successfully interviewed and those who were excluded (*p* = 0.192).

Compared to 2018–2019, general features of RSV‐infected patients at the same severity of condition were similar in 2020–2021, with the exception of age distribution and proportion of insured patients (Table S1). The mean direct medical cost during hospitalization for non‐severe RSV cases in 2018–2019 was 946.4 US$ (95% CI: 911.9–980.9 US$), and for severe cases, it was 6984.4 US$ (95% CI: 4352.7–9616.0 US$). Thus, both the values did not vary significantly in the two study periods. However, the sum of direct medical cost during hospitalization for patients without underlying conditions accounted for only 73.6% of total direct medical cost during hospitalization for all 386 RSV‐infected patients enrolled in 2018–2019, lower than 91.2% in 2020–2021.

### 3.4 Factors associated with cost of RSV hospitalization

Univariate and multivariate analyses of combined direct medical cost during hospitalization data for 2018–2019 and 2020–2021 revealed that patients at younger age, development of severe illness, and having at least one complication during the episode were associated with higher direct medical cost during hospitalization. The cost also increased with the extension of hospital length of stay (LOS) (Table S4).

Multivariate analysis of the data from 170 successfully interviewed patients revealed that younger age and longer LOS were associated with higher direct medical cost. Longer LOS was also the only factor associated with higher direct non‐medical cost and total cost. In our study, no particular factor was found to be related to indirect cost (not shown in tables).

### 3.5 Sensitivity analysis

During the telephone survey, 48.8% (83/170) of caregivers were unable to recall the exact direct medical cost prior to hospitalization, direct non‐medical cost, or indirect cost for at least one item and provided ranges instead. The lower and upper limits of the cost ranges were used in the sensitivity analyses (Tables S5–1 and S5–2). When the lower‐limits of the cost ranges were used, the mean total cost decreased by 3.3% compared to the analysis using a midrange value (1258.7 US$ vs. 1301.4 US$); when the upper‐limits were used, the cost increased by 4.8% (1363.8 US$ vs. 1301.4 US$).

### 3.6 HRQoL

Table 3 summarizes the results of the HRQoL survey and estimates of QALY loss per episode for the 170 successfully interviewed patients. The average total PedsQL™ score for 170 patients was 64.8 (95% CI: 63.5–66.0) with a mapped EQ‐5D‐Y utility of 0.8 (0.7–0.8). The estimated QALY loss per RSV episode was 8.9 × 10^−3^ (95% CI: 7.9 × 10^−3^–9.9 × 10^−3^). There was no significant difference in the QALY loss between patients aged 0–5 and 6–11 months (Table S2). Patients aged 0–11 months suffered more QALY loss than older age groups (*p* = 0.038). There was no significant difference in the QALY loss between patients with and without underlying conditions.

**TABLE 3:** Health‐related quality of life for non‐severe RSV‐infected inpatients.

Characteristics	PedsQL™ quality‐of‐life score			Mapped EQ‐5D‐Y utility score			QALY loss × 10^−3^		
	Mean (95% CI)	Median (IQR)	*p*‐Value	Mean (95% CI)	Median (IQR)	*p*‐Value	Mean (95% CI)	Median (IQR)	*p*‐Value
Overall	64.8 (63.5–66.0)	65.7 (60.4–71.5)		0.8 (0.7–0.8)	0.8 (0.7–0.9)		8.9 (7.9–9.9)	7.1 (5.1–10.3)	
Age group			0.025			0.170			0.072
0–11 months	63.7 (62.1–65.3)	64.6 (58.3–70.8)		0.8 (0.7–0.8)	0.8 (0.7–0.9)		9.7 (8.3–11.1)	7.9 (5.3–11.3)	
12–23 months	65.6 (63.1–68.0)	66.0 (61.8–71.5)		0.8 (0.7–0.8)	0.8 (0.7–0.9)		7.5 (6.2–8.9)	6.0 (4.9–8.5)	
24–59 months	67.8 (64.8–70.8)	69.1 (64.5–75.0)		0.8 (0.8–0.8)	0.8 (0.8–0.9)		8.0 (5.4–10.6)	6.3 (4.1–8.2)	
Gender			0.197			0.292			0.069
Male	64.4 (62.9–65.9)	65.3 (60.7–69.9)		0.8 (0.7–0.8)	0.8 (0.7–0.8)		9.2 (7.9–10.6)	7.8 (5.7–10.6)	
Female	65.4 (63.2–67.6)	67.4 (60.4–72.3)		0.8 (0.7–0.8)	0.8 (0.7–0.9)		8.3 (6.9–9.7)	6.4 (4.7–9.5)	
Underlying conditions			0.466			0.385			0.773
No	65.0 (63.7–66.2)	66.0 (60.4–71.5)		0.8 (0.7–0.8)	0.8 (0.7–0.9)		8.9 (7.8–9.9)	7.1 (5.1–10.5)	
Yes	62.2 (54.7–69.8)	63.9 (60.4–68.6)		0.7 (0.6–0.8)	0.7 (0.7–0.8)		8.5 (5.4–11.6)	8.7 (5.8–9.0)	

Abbreviations: CI, confidence interval; IQR, interquartile range; QALY, quality adjusted life years.

## 4 DISCUSSION

This prospective study was conducted in Central China to estimate the total cost and assess the HRQoL associated with RSV‐infected pediatric inpatients by extracting data from HIS and conducting telephone surveys. Our study found that RSV related hospitalization is a key health concern for patients aged 0–59 months, particular for <1‐year‐olds, causing substantial economic burden. The mean cost per RSV episode for inpatients was 1301.4 US$ (95% CI: 1230.7–1372.0 US$). The estimated QALY loss per RSV episode was 8.9 × 10^−3^ (95% CI: 7.9 × 10^−3^–9.9 × 10^−3^).

General characteristics of patients and direct medical cost during hospitalization were comparable in 2018–2019 and 2020–2021 for the patients with similar severities in condition. Moreover, no significant difference in the general characteristics of the patients was observed between those who were successfully interviewed and those who were excluded in 2020–2021. Hence, we can assume that the results of this study would well depict the scenario for the RSV‐infected patients admitted to Henan Children's Hospital in other years also.

Our study found that the self‐paid portion of the total cost accounted for a non‐negligible proportion of the per capita disposable income of Henan province—about 24.6% of 4156 US$. Similarly, the total cost makes up 14.1% of GDP per capita of Henan province or 2.4 times the national health expenditure per capita.^26^, ^27^ A study conducted in Jiangsu province of eastern China from 2005 to 2009 found that the mean cost of each RSV‐related hospitalization was 571.8 US$, equivalent to 11.3% of local average per capita annual income in 2010.^10^ The United States evaluated the pooled direct medical cost of RSV‐related hospitalization to be 4017 US$ per episode (adjusted to 2020) for patients aged 0–59 months without high risks,^28^ making up about 8.4% of disposable personal income per capita of that year. In the developed countries of Organization for Economic Co‐operation and Development (OECD), the cost of treatment for RSV‐infected inpatients was also higher than the total health expenditure per capita.^5^ In developing countries like Bangladesh, the median out‐of‐pocket expense for RSV‐related hospitalization represented 24% of the monthly household income.^29^ In Malawi, the cost of each RSV hospitalization was about 15.8% of its GDP per capita.^12^ Despite of the vast heterogeneity in the results due to differences in the socioeconomic conditions or insurance schemes across the countries, the above studies unambiguously establish that RSV‐related hospitalization poses a heavy economic burden on both the patient‐family and the society.

Younger age was found to be associated with increased cost of RSV‐related hospitalization in this study, consistent with the findings from Demont et al. showing infants <1 year old represented 80% of the economic burden of RSV among children aged <5 years.^30^ Additionally, this study also found that significantly higher direct medical cost during hospitalization was associated with patients having underlying conditions (e.g., preterm birth). This finding is also consistent with previous studies.^31^, ^32^, ^33^, ^34^, ^35^, ^36^ However, it should be highlighted that the majority of RSV‐infected children did not have any underlying conditions (“otherwise healthy”). Together, such patients consumed 73.6%–92.9% of the total cost spent; this finding is also consistent with previous studies.^35^, ^37^, ^38^ For example, Bowser et al. evaluated that full‐term infants accounted for 70%–82% of the total RSV hospitalization cost among US children aged 0–59 months.^28^ Considering the large population and the high incidence of RSV in Chinese children,^39^ total cost for otherwise healthy cases should be higher than those with underlying conditions from the perspective of the entire society. We believe that future immunization strategy covering all young children, irrespective of the presence of risk factors, could successfully reduce the economic burden of RSV.

Only limited number of studies were present previously assessing the HRQoL of RSV‐infected children. Glaser et al. estimated the QALY loss among hospitalized premature infants to be 0.0173 per episode by direct observation^40^ whereas using a model, Hodgson et al. predicted a QALY loss of 3.823 × 10^−3^ per RSV episode for children under 5 years of age who sought health care.^41^ Villamil et al. reported 20 disability‐adjusted life years (DALYs)/1000 person‐year in Colombian children aged <2 years due to RSV bronchiolitis.^42^ In our study, the QALY loss of premature infants did not differ significantly from that of other children and was lower than the QALY loss reported by Glaser et al. However, it was higher than the value reported by Hodgson et al. as our study did not involve outpatients for whom less QALY loss is expected. Other potential factors producing the differences could be selection of different measuring tools, difference in target time period of evaluation, and the discrepancies arising due to the collection of data from parents/caregivers rather than direct measurement of QoL of the young children.^40^

Our study has several limitations. Firstly, telephone survey was carried out at least half a month after the discharge making the recall bias inevitable. Secondly, the severe cases could not be reached by the telephone survey preventing us from evaluating direct non‐medical, indirect cost, and HRQoL for severely ill inpatients. Thirdly, some cost might not be taken into account in this analyses. For instance, the interview did not inquire potential medical cost incurred after hospitalization, and the cost of ambulance services was not included in direct medical cost (one case reported being transferred to the hospital by ambulance). The factors above may lead to an underestimation of the overall economic burden during the whole course of RSV infection. Besides, the utility score for fully healthy children was assumed to be 1.0 in the HRQoL study^23^ undermining the fact that children might not be “fully healthy” before infection. This could overestimate the QALY loss in our study. Also, the mapping algorithm used in our study was developed based on data from children aged 11–15 years. As a result, the estimated utility of younger children with different clinical characteristics might have a degree of uncertainty. Finally, the data used in the present study were collected for patients of only one hospital in one province. Therefore, the results are unlikely to represent the true scenario of other areas in the diverse healthcare settings of China.

## 5 CONCLUSIONS

Our study revealed that RSV‐allied hospitalization causes high economic and health burden, particularly for infants <1 year old. The findings are not only valuable for a comprehensive understanding the economic and health burden of RSV‐related hospitalization in China but also useful for prioritizing the public health interventions and optimizing the allocation of limited health resources. Further studies are required for obtaining deeper and broader insights into the economic burden and HRQoL associated to RSV infection in China.

## AUTHOR CONTRIBUTIONS

**Lingshuang Ren**: Conceptualization; investigation; formal analysis; writing—original draft. **Lidan Cui**: Project administration; resources. **Qianli Wang**: Project administration; writing—review and editing. **Liujiong Gao**: Project administration. **Meng Xu**: Investigation. **Meng Wang**: Investigation. **Qianhui Wu**: Formal analysis. **Jinxin Guo**: Formal analysis. **Li Lin**: Project administration. **Yuxia Liang**: Investigation. **Nuolan Liu**: Investigation. **Yibing Cheng**: Project administration; supervision. **Juan Yang**: Methodology; writing—review and editing. **Hongjie Yu**: Conceptualization; supervision; writing—review and editing.

## CONFLICT OF INTEREST STATEMENT

HY has received research funding from Sanofi, Shanghai Roche Pharmaceutical Company, GlaxoSmithKline (China) Investment Co., Ltd., Yichang HEC Changjiang Pharmacertical Co., Ltd., SINOVAC Biotech Ltd. and Shenzhen Sanofi Pasteur Biological Products Co., Ltd.. All other authors declare no competing interests.

### PEER REVIEW

The peer review history for this article is available at https://www.webofscience.com/api/gateway/wos/peer-review/10.1111/irv.13180.

## ETHICS STATEMENT

The study was approved by School of public health, Fudan University (IRB No. 2018‐06‐0686 and IRB No.2020‐12‐0864) and Research Ethics Commission of Henan Children's Hospital.

## Supporting information


**Table S1.** Comparison of RSV‐infected inpatients between two study periods.
**Table S2.** The number, cost and health‐related quality of life of RSV‐infected inpatients under 1 year of age.
**Table S3.** Direct medical cost during hospitalization for all 261 RSV‐infected inpatients.
**Table S4.** Factors associated with direct medical cost during hospitalization.
**Table S5–1.** Sensitivity analysis for cost of RSV‐infected inpatients (lower limits).
**Table S5–2.** Sensitivity analysis for cost of RSV‐infected inpatients (upper limits).
**Figure S1.** Composition of total cost for patients hospitalized with RSV.

**Data S1.** Supporting information

## Data Availability

The data are not publicly available due to ethical restrictions and privacy. De‐identified individual data that supports the findings of this study are available from the corresponding authors on reasonable request. The data requestor will need to sign a data access agreement.
