# Supplementary material for: Cost and health‐related quality of life for children hospitalized with respiratory syncytial virus in Central China
Source: Influenza Other Respir Viruses. 2023 Aug 17;17(8):e13180. doi: 10.1111/irv.13180 (PMC10495873; doi:10.1111/irv.13180)
Supplement: Supplementary file 1 — Table S1. Comparison of RSV‐infected inpatients between two study periods. Table S2. The number, cost and health‐related quality of life of RSV‐infected inpatients under 1 year of age. Table S3. Direct medical cost during hospitalization for all 261 RSV‐infected inpatients. Table S4. Factors associated with direct medical cost during hospitalization. Table S5–1. Sensitivity analysis for cost of RSV‐infected inpatients (lower limits). Table S5–2. Sensitivity analysis for cost of RSV‐infected inpatients (upper limits). Figure S1. Composition of total cost for patients hospitalized with RSV. [file IRV-17-e13180-s001.docx]

**Supplementary materials**

**Cost and Health-Related Quality of Life for Children Hospitalized with Respiratory Syncytial Virus in Central China**

Lingshuang Ren, Lidan Cui, Qianli Wang, Liujiong Gao, Meng Xu, Meng Wang, Qianhui Wu, Jinxin Guo, Li Lin, Yuxia Liang, Nuolan Liu, Yibing Cheng, Juan Yang, Hongjie Yu

# METHOD

**Mapping algorithm**

The mapping algorithm for converting PedsQL^TM^ score into EQ-5D-Y Utility score is shown below. The model was developed by Khan et al.^1^

EQ-5D-Y Utility score = 0.009127 × PF + 0.006611 × EF + 0.005705 × SF + 0.006011 × (CogF or SchF) +0.000020 × PF^2^ - 0.000048 × EF^2^ + 0.000011 × SF^2^ - 0.000017 × (CogF or SchF)^2^ - 0.000004 × PF ×EF - 0.000055 × PF × SF - 0.000066 × PF × (CogF or SchF) - 0.000009 × EF × SF + 0.000059 × EF × (CogF or SchF) - 0.000027 × SF × (CogF or SchF) - 0.428496

Where PF, EF, SF, CogF and SchF denote the average scores of the following dimensions in PedsQL respectively: physical functioning, emotional functioning, social functioning, cognitive functioning and school functioning.

**Reference**

1. Khan KA, Petrou S, Rivero-Arias O, Walters SJ, Boyle SE. Mapping EQ-5D utility scores from the PedsQL™ generic core scales. *Pharmacoeconomics.* 2014;32(7):693-706. doi:[10.1007/s40273-014-0153-y](https://doi.org/10.1007/s40273-014-0153-y)

# TABLES

**Table S1. Comparison of RSV-infected inpatients between two study periods**

| Characteristic | Non–severe RSV cases | | |  | Severe RSV cases | | |
| --- | --- | --- | --- | --- | --- | --- | --- |
|  | **2018–2019** | **2020–2021** | ***p* value** |  | **2018–2019** | **2020–2021** | ***p* value** |
|  | **(n =356)** | **(n =256)** |  |  | **(n =30)** | **(n =5)** |  |
| **Direct medical cost during hospitalization** | 946.4 (911.9–980.9) | 945.1 (911.3–978.8) | 0.662 |  | 6984.4 (4352.7–9616.0) | 7207.8 (82.7–14333.0) | 0.631 |
| Out-of-pocket portion | 698.8 (667.5–730.1) | 676.4 (641.8–711.0) | 0.214 |  | 3865.5 (2328.8–5402.2) | 5293.0 (-2688.4–13274.4) | 0.631 |
| Reimbursable portion | 247.6 (220.0–275.2) | 268.6 (243.4–293.9) | 0.085 |  | 3118.8 (1339.5–4898.2) | 1914.8 (-837.8–4667.3) | 0.684 |
| **Age; month, median [IQR]** | 4.3 [2.3–12.4] | 7.5 [2.7–18.0] | **<0.001** |  | 5.6 [2.7–14.2] | 3.0 [2.5–5.0] | 0.448 |
| **Age group** |  |  | **<0.001** |  |  |  | >0.999 |
| 0–11 months | 265 (74.4) | 148 (57.8) |  |  | 21 (70.0) | 4 (80.0) |  |
| 12–23 months | 52 (14.6) | 62 (24.2) |  |  | 5 (16.7) | 1 (20.0) |  |
| 24–60 months | 39 (11.0) | 46 (18.0) |  |  | 4 (13.3) | 0 (0) |  |
| **Gender** |  |  | 0.779 |  |  |  | 0.052 |
| Male | 214 (60.1) | 151 (59.0) |  |  | 21 (70.0) | 1 (20.0) |  |
| Female | 142 (39.9) | 105 (41.0) |  |  | 9 (30.0) | 4 (80.0) |  |
| **Insurance** |  |  | **<0.001** |  |  |  | 0.640 |
| No | 150 (42.1) | 189 (73.8) |  |  | 9 (30.0) | 2 (40.0) |  |
| Yes | 206 (57.9) | 67 (26.2) |  |  | 21 (70.0) | 3 (60.0) |  |
| **Underlying condition** |  |  | 0.085 |  |  |  | >0.999 |
| No | 318 (89.3) | 239 (93.4) |  |  | 19 (63.3) | 3 (60.0) |  |
| Yes | 38 (10.7) | 17 (6.6) |  |  | 11 (36.7) | 2 (40.0) |  |
| **Complications** |  |  | >0.999 |  |  |  | >0.999 |
| No | 4 (1.1) | 3 (1.2) |  |  | 0 (0) | 0 (0) |  |
| Yes | 352 (98.9) | 253 (98.8) |  |  | 30 (100) | 5 (100) |  |
| **Length of hospital stay, day, median [IQR]** | 6.0 [5.0–7.0] | 6.0 [5.0–7.0] | 0.636 |  | 8.5 [4.0-10.8] | 16.0 [12.0-16.0] | **0.011** |

Abbreviations: IQR, interquartile range.

Note: Figures are mean (95% CI) of cost or numbers of patients (%) unless stated otherwise. Characteristics are compared between 2018–2019 and 2020–2021. A *p* value of <0.05 was considered statistically significant.

**Table S2. The number, cost and health-related quality of life of RSV-infected inpatients under 1 year of age**

|  | 0–5 months | 6–11 months |
| --- | --- | --- |
| **Number of patients (%)** |  |  |
| Overall | 115/261 (44.1) | 37/261 (14.2) |
| Interviewed | 81/170 (47.6) | 26/170 (15.3) |
| Excluded | 34/91 (37.4) | 11/91 (12.1) |
| **Cost of patients successfully interviewed (mean, 95% CI)** |  |  |
| Direct medical cost | 1180.4 (1092.3–1268.5) | 1066.3 (891.5–1241.1) |
| Direct non-medical cost | 92.1 (82.1–102.1) | 88.7 (74.9–102.6) |
| Indirect cost | 140.9 (94.3–187.4) | 234.6 (117.3–351.9) |
| **Health-related quality of life successfully interviewed (mean, 95% CI)** |  |  |
| PedsQL^TM^ quality-of-life score | 63.9 (62.0–65.8) | 62.9 (59.7–66.1) |
| Mapped EQ-5D-Y utility score | 0.8 (0.7–0.8) | 0.7 (0.7–0.8) |
| QALY loss×10^-3^ | 9.7 (7.9–11.4) | 9.0 (7.5–10.5) |

**Table S3. Direct medical cost during hospitalization for all 261 RSV-infected inpatients**

| Characteristics | Direct medical cost during hospitalization | *p* value |
| --- | --- | --- |
| **Direct medical cost during hospitalization** | 1065.1 (925.0–1205.1) |  |
| Out-of-pocket portion | 764.9 (636.2–893.6) |  |
| Reimbursable portion | 300.2 (250.2–350.1) |  |
| **Age group** |  | **<0.001** |
| 0–11 months | 1221.0 (987.1–1455) |  |
| 12–23 months | 846.0 (774.7–917.2) |  |
| 24–59 months | 849.6 (728.6–970.6) |  |
| **Gender** |  | 0.707 |
| Male | 1005.5 (900.8–1110.2) |  |
| Female | 1148.1 (843.8–1452.5) |  |
| **Insurance** |  | 0.936 |
| No | 1227.0 (761.1–1692.9) |  |
| Yes | 1006.9 (911.9–1101.8) |  |
| **Underlying condition** |  | **0.007** |
| No | 1047.9 (898.9–1197.0) |  |
| Yes | 1283.1 (956.5–1609.6) |  |
| **Severe illness** |  | **<0.001** |
| No | 945.1 (911.3–978.8) |  |
| Yes | 7207.8 (82.7–14333.0) |  |
| **Complications** |  | **0.032** |
| No | 651.7 (246.8–1056.5) |  |
| Yes | 1069.9 (928.3–1211.4) |  |
| **Telephone survey** |  | 0.192 |
| Interviewed | 959.3 (921.4–997.3) |  |
| Excluded^†^ | 1262.6 (865.5–1659.7) |  |
| Non-severe | 916.9 (849.2–984.6) |  |

Note: Figures are mean (95% CI) of cost. This table describes direct medical cost during hospitalization of all 261 RSV-infected patients enrolled in 2020–2021.

^†^The reasons for exclusion include unreachable, rejecting to participate or failed to recall cost of the RSV episode.

**Table S4. Factors associated with direct medical cost during hospitalization**

| Characteristics | Univariate analysis | |  | Multivariate analysis | |
| --- | --- | --- | --- | --- | --- |
|  | **Coefficient (95% CI)** | ***p* value** |  | **Coefficient (95% CI)** | ***p* value** |
| **Age group** |  |  |  |  |  |
| 0–11 months | Reference | - |  | Reference | - |
| 12–23 months | -0.13 (-0.23–-0.02) | **0.017** |  | -0.07 (-0.12–-0.02) | **0.006** |
| 24–60 months | -0.17 (-0.28–-0.05) | **0.005** |  | -0.11 (-0.16–-0.05) | **<0.001** |
| **Underlying conditions** |  |  |  |  |  |
| No | Reference | - |  | Reference | - |
| Yes | 0.37 (0.25–0.5) | **<0.001** |  | 0.05 (-0.02–0.11) | 0.144 |
| **Severe illness** |  |  |  |  |  |
| No | Reference | - |  | Reference | - |
| Yes | 1.7 (1.59–1.82) | **<0.001** |  | 1.3 (1.2–1.41) | **<0.001** |
| **Complications** |  |  |  |  |  |
| No | Reference | - |  | Reference | - |
| Yes | 0.37 (0.32–0.42) | **<0.001** |  | 0.07 (0.04–0.1) | **<0.001** |
| **Hospital length of stay** | 0.13 (0.12–0.14) | **<0.001** |  | 0.09 (0.08–0.1) | **<0.001** |

Abbreviations: CI, confidence interval.

Note: 647 subjects under 5 years old detected RSV positive in two years were included into analysis: 386 in 2018–2019, and 261 in 2020–2021. The cost was logarithmically transformed before performing GLM analysis. Characteristics with *p* value above 0.10 in univariate analyses were excluded.

**Table S5−1. Sensitivity analysis for cost of RSV-infected inpatients (lower limits)**

| Characteristics | Direct medical cost | *p* value |  | Direct non-medical cost | *p* value |  | Indirect cost | *p* value |
| --- | --- | --- | --- | --- | --- | --- | --- | --- |
| **Overall** | 1042.1 (985.6–1098.5) |  |  | 60.3 (54.7–65.8) |  |  | 156.3 (124.0–188.7) |  |
| **Age group** |  | **<0.001** |  |  | **0.024** |  |  | 0.661 |
| 0–11 months | 1142.5 (1065–1219.9) |  |  | 61.1 (47.7–74.5) |  |  | 230.3 (113.7–346.9) |  |
| 12–23 months | 884.1 (784.8–983.4) |  |  | 48.6 (40.2–56.9) |  |  | 143.4 (66.1–220.6) |  |
| 24–59 months | 857.7 (795.5–919.8) |  |  | 52.4 (39.6–65.2) |  |  | 174.3 (92.5–256.1) |  |
| **Gender** |  | 0.662 |  |  | 0.352 |  |  | 0.286 |
| Male | 1060.1 (979.9–1140.3) |  |  | 58.2 (50.9–65.6) |  |  | 159.0 (120.1–197.9) |  |
| Female | 1014.4 (939.2–1089.5) |  |  | 63.3 (54.8–71.9) |  |  | 152.2 (94.6–209.8) |  |
| **Insurance** |  | 0.437 |  |  | 0.563 |  |  | 0.150 |
| No | 1112.3 (960.8–1263.7) |  |  | 59.3 (47.1–71.4) |  |  | 150.0 (69.6–230.4) |  |
| Yes | 1017.5 (961.6–1073.4) |  |  | 60.6 (54.3–66.9) |  |  | 158.6 (124.3–192.8) |  |
| **Underlying conditions** |  | 0.057 |  |  | **0.045** |  |  | 0.478 |
| No | 1034.0 (975.0–1093.0) |  |  | 59.1 (53.4–64.9) |  |  | 151.9 (119.7–184.1) |  |
| Yes | 1158.3 (949.1–1367.6) |  |  | 76.3 (53.7–98.9) |  |  | 220.6 (7.2–434.0) |  |
| **Complications** |  | 0.095 |  |  | 0.263 |  |  | 0.885 |
| No | 803.0 (503.4–1102.6) |  |  | 36.7 (-66.7–140.1) |  |  | 138.6 (-213.5–490.7) |  |
| Yes | 1044.9 (987.9–1101.9) |  |  | 60.5 (54.9–66.1) |  |  | 156.6 (123.8–189.3) |  |

Note: Figures are mean (95% CI) of cost.

**Table S5−2. Sensitivity analysis for cost of RSV-infected inpatients (upper limits)**

| Characteristics | Direct medical cost | *p* value |  | Direct non-medical cost | *p* value |  | Indirect cost | *p* value |
| --- | --- | --- | --- | --- | --- | --- | --- | --- |
| **Overall** | 1068.8 (1010.7–1126.9) |  |  | 126.5 (115.1–137.9) |  |  | 168.5 (130.9–206.0) |  |
| **Age group** |  | **<0.001** |  |  | **0.005** |  |  | 0.196 |
| 0–11 months | 1076.1 (900.7–1251.5) |  |  | 138.3 (99.4–177.2) |  |  | 238.8 (120.2–357.5) |  |
| 12–23 months | 923.1 (812.3–1033.9) |  |  | 105.5 (81.7–129.4) |  |  | 150.5 (70.4–230.7) |  |
| 24–59 months | 892.9 (821.1–964.6) |  |  | 108.8 (89.1–128.5) |  |  | 175.8 (94.0–257.6) |  |
| **Gender** |  | 0.511 |  |  | 0.924 |  |  | 0.275 |
| Male | 1089.5 (1007.8–1171.1) |  |  | 126.8 (111.6–142.1) |  |  | 168.7 (126.0–211.3) |  |
| Female | 1037.0 (957.7–1116.3) |  |  | 126.0 (108.4–143.6) |  |  | 168.2 (97.6–238.8) |  |
| **Insurance** |  | 0.556 |  |  | 0.563 |  |  | 0.162 |
| No | 1129.4 (978.1–1280.6) |  |  | 126.5 (98.8–154.1) |  |  | 166.3 (76.8–255.8) |  |
| Yes | 1047.6 (988.6–1106.7) |  |  | 126.5 (114.2–138.8) |  |  | 169.2 (128.5–209.9) |  |
| **Underlying conditions** |  | 0.109 |  |  | **0.027** |  |  | 0.462 |
| No | 1062.4 (1001.6–1123.2) |  |  | 124.3 (112.4–136.2) |  |  | 163.5 (125.6–201.4) |  |
| Yes | 1161.1 (952.7–1369.4) |  |  | 158.0 (115.4–200.7) |  |  | 240.8 (19.3–462.2) |  |
| **Complications** |  | 0.172 |  |  | 0.206 |  |  | 0.802 |
| No | 803.0 (503.4–1102.6) |  |  | 77.1 (-268.3–422.4) |  |  | 145.9 (-300.1–591.9) |  |
| Yes | 1072.0 (1013.3–1130.6) |  |  | 127.1 (115.5–138.6) |  |  | 168.7 (130.8–206.7) |  |

Note: Figures are mean (95%CI) of cost.

# FIGURE


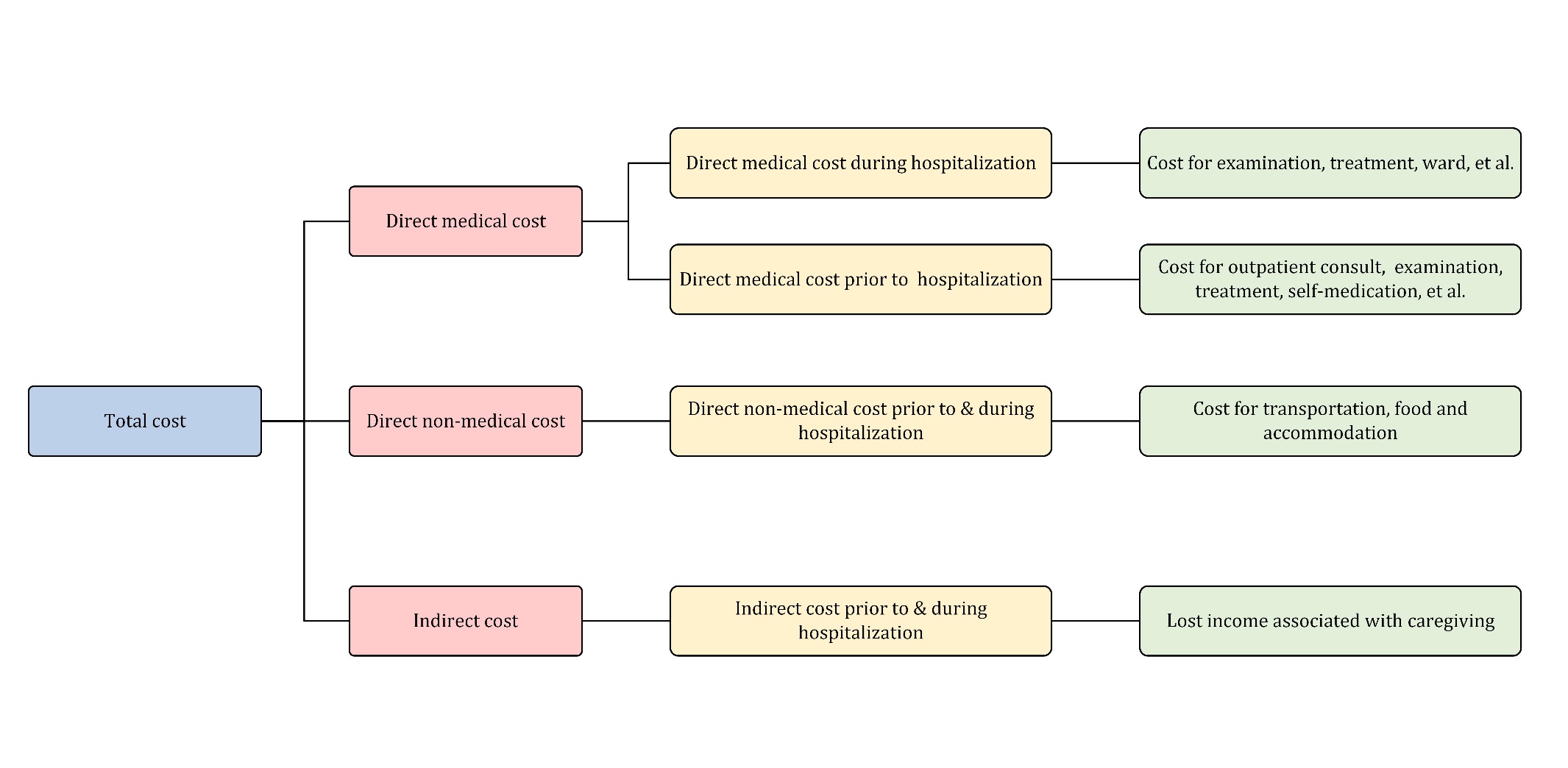


**Figure S1. Composition of total cost for patients hospitalized with RSV**
