# Supplementary material for: Cost and health‐related quality of life for children hospitalized with respiratory syncytial virus in Central China
Source: Influenza Other Respir Viruses. 2023 Aug 17;17(8):e13180. doi: 10.1111/irv.13180 (PMC10495873; doi:10.1111/irv.13180)
Supplement: Supplementary file 2 — Data S1. Supporting information [file IRV-17-e13180-s002.docx]

**Telephone Interview Questionnaire (brief version)**

| **Dimensions** | | **Items** | | **Options or answers** |
| --- | --- | --- | --- | --- |
| Information to be verified before the interview | | i. Are you the contact person of xxx（the name of the patient）？ | | 1) Yes; 2) No |
|  |  | ii. What’s the relationship between you and the patient? | | 1) parents; 2) grandparents; 3) other |
|  |  | iii. Who mainly cared for the patient during hospitalization? | | 1) parents; 2) grandparents; 3) other |
| Disease-related information | | 1.Did the patient still have any symptoms or signs at discharge? | | 1) Yes; 2) No |
|  |  | 2.How long did it take for all the symptom and signs to disappeared after discharge? | | ____ days |
| Direct medical cost prior to hospitalization | | 3.Did the patient seek for medical service in any healthcare facilities before hospitalization? | | 1) Yes; 2) No |
|  |  | 4.How much did the medical service cost for the patient in these healthcare facilities before hospitalization？ | | yuan |
| Direct non-medical cost prior to hospitalization | | 5.What type of transportation did it take the patient to seek for medical service before hospitalization? | | 1) self-driving; 2) taxi; 3) public transport; 4) (electric) bicycle; 5) others |
|  |  | 6.How much it cost for the patient spend on transportation for the medical service before hospitalization？ | | yuan |
|  |  |  |  | If the interviewee drove to hospitals and cannot estimate the cost, please ask him/her to tell the distance and between home and the hospital: _____kilometers, as well as travelling times: ______times |
| Direct non-medical cost during hospitalization | | 7.How many persons accompanied and cared for the patient during hospitalization, i.e., the number of caregivers? | | ____persons |
|  |  | 8.What type of transportation did the patient and caregivers take for hospitalization? | | 1) self-driving; 2) taxi; 3) public transport; 4) (electric) bicycle; 5) others |
|  |  | 9.How much did the patient and caregivers spend on transportation during the patient's hospitalization？ | | yuan |
|  |  |  |  | If the interviewee drove to hospitals and cannot estimate the cost, please ask him/her to tell the distance and between home and the hospital: _____kilometers, as well as travelling times: ______times |
|  |  | 10.How much did the patient and caregivers spend on meals during the patient's hospitalization? | | yuan in total or _____yuan/day |
|  |  | 11.How much did the caregivers spend on accommodation during the patient's hospitalization? | | yuan in total or _____yuan/day |
| Indirect cost | | 12.How many days did the caregivers ask for leave due to accompanying care? | | _____days |
| Health-related quality of life |  | **PedsQL^TM^ Infant Scales (for patients aged up to 24 months)** | **PedsQL^TM^ 4.0 Generic Core Scales (for patients aged 2-5 years)** |  |
|  | Physical functioning | 1. Low energy level | 1.Walking | 1) Never; 2) Almost never; 3) Sometimes; 4) Often; 5) Almost Always |
|  |  | 2. Difficulty participating in active play | 2.Running | 1) Never; 2) Almost never; 3) Sometimes; 4) Often; 5) Almost Always |
|  |  | 3. Having hurts or aches | 3.Participating in active play or exercise | 1) Never; 2) Almost never; 3) Sometimes; 4) Often; 5) Almost Always |
|  |  | 4. Feeling tired | 4.Lifting something heavy | 1) Never; 2) Almost never; 3) Sometimes; 4) Often; 5) Almost Always |
|  |  | 5. Being lethargic | 5.Bathing | 1) Never; 2) Almost never; 3) Sometimes; 4) Often; 5) Almost Always |
|  |  | 6. Resting a lot | 6.Helping to pick up his or her toys | 1) Never; 2) Almost never; 3) Sometimes; 4) Often; 5) Almost Always |
|  |  | 7. Feeling too tired to play (only aged 13-24 months answer) | 7.Having hurts or aches | 1) Never; 2) Almost never; 3) Sometimes; 4) Often; 5) Almost Always |
|  |  | 8. Difficulty walking (only aged 13-24 months answer) | 8.Low energy level | 1) Never; 2) Almost never; 3) Sometimes; 4) Often; 5) Almost Always |
|  |  | 9. Difficulty running a short distance without falling (only aged 13-24 months answer) |  | 1) Never; 2) Almost never; 3) Sometimes; 4) Often; 5) Almost Always |
|  | Emotional functioning | 1. Feeling afraid or scared | 1.Feeling afraid or scared | 1) Never; 2) Almost never; 3) Sometimes; 4) Often; 5) Almost Always |
|  |  | 2. Feeling angry | 2.Feeling sad or blue | 1) Never; 2) Almost never; 3) Sometimes; 4) Often; 5) Almost Always |
|  |  | 3. Crying or fussing when left alone | 3.Feeling angry | 1) Never; 2) Almost never; 3) Sometimes; 4) Often; 5) Almost Always |
|  |  | 4. Difficulty soothing himself/herself when upset | 4.Trouble sleeping | 1) Never; 2) Almost never; 3) Sometimes; 4) Often; 5) Almost Always |
|  |  | 5. Difficulty falling asleep | 5.Worrying | 1) Never; 2) Almost never; 3) Sometimes; 4) Often; 5) Almost Always |
|  |  | 6. Crying or fussing while being cuddled |  | 1) Never; 2) Almost never; 3) Sometimes; 4) Often; 5) Almost Always |
|  |  | 7. Feeling sad |  | 1) Never; 2) Almost never; 3) Sometimes; 4) Often; 5) Almost Always |
|  |  | 8. Difficulty being soothed when picked up or held |  | 1) Never; 2) Almost never; 3) Sometimes; 4) Often; 5) Almost Always |
|  |  | 9. Difficulty sleeping mostly through the night |  | 1) Never; 2) Almost never; 3) Sometimes; 4) Often; 5) Almost Always |
|  |  | 10. Crying a lot |  | 1) Never; 2) Almost never; 3) Sometimes; 4) Often; 5) Almost Always |
|  |  | 11. Feeling cranky |  | 1) Never; 2) Almost never; 3) Sometimes; 4) Often; 5) Almost Always |
|  |  | 12. Difficulty taking naps during the day |  | 1) Never; 2) Almost never; 3) Sometimes; 4) Often; 5) Almost Always |
|  | Social functioning | 1. Not smiling at others | 1.Playing with other children | 1) Never; 2) Almost never; 3) Sometimes; 4) Often; 5) Almost Always |
|  |  | 2. Not laughing when tickled | 2.Other kids not wanting to play with him or her | 1) Never; 2) Almost never; 3) Sometimes; 4) Often; 5) Almost Always |
|  |  | 3. Not making eye contact with a caregiver | 3.Getting teased by other children | 1) Never; 2) Almost never; 3) Sometimes; 4) Often; 5) Almost Always |
|  |  | 4. Not laughing when cuddled | 4.Not able to do things that the children his or her age can do | 1) Never; 2) Almost never; 3) Sometimes; 4) Often; 5) Almost Always |
|  |  | 5. Being uncomfortable around other children (only aged 13-24 months answer) | 5.Keeping up when playing with other children | 1) Never; 2) Almost never; 3) Sometimes; 4) Often; 5) Almost Always |
|  | Cognitive / school functioning | 1. Not imitating caregivers' actions | 1.Doing the same school activities as peers | 1) Never; 2) Almost never; 3) Sometimes; 4) Often; 5) Almost Always |
|  |  | 2. Not imitating caregivers' facial expressions | 2.Missing school/daycare because of not feeling well | 1) Never; 2) Almost never; 3) Sometimes; 4) Often; 5) Almost Always |
|  |  | 3. Not imitating caregivers' sounds | 3.Missing school/daycare to go to the doctor or | 1) Never; 2) Almost never; 3) Sometimes; 4) Often; 5) Almost Always |
|  |  | 4. Not able to fix his/her attention on objects |  | 1) Never; 2) Almost never; 3) Sometimes; 4) Often; 5) Almost Always |
|  |  | 5. Not imitating caregivers' speech (only aged 13-24 months answer) |  | 1) Never; 2) Almost never; 3) Sometimes; 4) Often; 5) Almost Always |
|  |  | 6. Difficulty pointing to his/her body parts when asked (only aged 13-24 months answer) |  | 1) Never; 2) Almost never; 3) Sometimes; 4) Often; 5) Almost Always |
|  |  | 7. Difficulty naming familiar objects (only aged 13-24 months answer) |  | 1) Never; 2) Almost never; 3) Sometimes; 4) Often; 5) Almost Always |
|  |  | 8. Difficulty repeating words (only aged 13-24 months answer) |  | 1) Never; 2) Almost never; 3) Sometimes; 4) Often; 5) Almost Always |
|  |  | 9. Difficulty keeping his/her attention on things (only aged 13-24 months answer) |  | 1) Never; 2) Almost never; 3) Sometimes; 4) Often; 5) Almost Always |
